# Filling the Gap: Establishing a Statewide Tick and Tick-Borne Pathogen Surveillance Program

**DOI:** 10.3390/insects17040414

**Published:** 2026-04-12

**Authors:** Kyndall C. Dye-Braumuller, Lídia Gual-Gonzalez, Emily Owens Pickle, Christopher Lee, Madeleine M. Meyer-Torelli, Chris L Evans, Jennifer G. Chandler, Rebecca T. Trout Fryxell, Melissa S. Nolan

**Affiliations:** 1 Institute for Infectious Disease Translational Research, University of South Carolina, Columbia, SC 29208, USA; 2 Department of Epidemiology and Biostatistics, Arnold School of Public Health, University of South Carolina, Columbia, SC 29208, USA; 3 Department of Public Health Sciences, Clemson University, Clemson, SC 29634, USA; 4 South Carolina Department of Public Health, Bureau of Environmental Health Services, 8500 Farrow Road, State Park Building 5 Room 509, Columbia, SC 29203, USA; 5 Department of Entomology and Plant Pathology, The University of Tennessee, Knoxville, TN 37996, USA

**Keywords:** ticks, surveillance, South Carolina, invasive species, tick-borne disease, citizen science

## Abstract

Individuals in the southeastern United States of America (USA) have an increasing risk of contracting a tick-borne disease. Land use changes, changing climate, and redistribution of both ticks and their hosts make systematic tick and tick-borne pathogen investigation crucial for public health protection. Prior to 2020, South Carolina had limited data on tick species distribution and tick infection rates. In this work, we describe establishment of a sustainable tick and tick-borne pathogen collaborative network for South Carolina. A major determinant of program success was sharing work effort between the University of South Carolina, the South Carolina Department of Public Health, and key partners including state park employees, local veterinarians, students, and volunteers. The program collected questing ticks from public lands and host-attached ticks from animal shelters. Amblyomma americanum was the most commonly collected tick, with highest density in South Carolina’s southern coastal region. A greater tick species diversity was seen in animal shelter collected versus questing ticks. Pathogen testing results yielded a high presence of *Rickettsia amblyommatis* among *Am. americanum* ticks with several other *Rickettsia* spp. detected including *Rickettsia parkeri*, Candidatus *R. andeanae*, *R. montanensis*, and *R. asembonensis*. Additional Rickettsiales detected included multiple Ehrlichia and Anaplasma species, with higher presence in the state’s northern region. Borrelia burgdorferi sensu stricto was detected in one questing Ixodes keiransi from the southern coastal region. The current report presents the initial steps for pathogen and tick species surveillance in South Carolina, providing successes and pitfalls as a model for other states and regions to establish similar efforts to improve national tick surveillance.

## Simple Summary

Ticks are becoming more common in the southeastern United States, and they may carry pathogens that make people and animals sick. In South Carolina, there has been limited information about where ticks are found and what diseases they may spread. To address this, we created a statewide program that brings together researchers, public health officials, and community partners like state parks and animal shelters to collect and study ticks. In the first year of the collaborative, statewide program, we found that the lone star tick was the most common species across the state, especially in coastal areas. Many ticks carried bacteria that can cause illness, although some detected pathogens are not yet fully understood. By building this collaborative program, we were able to gather important information about tick populations and disease risk that can inform future public health programs.

## 1. Introduction

From 2004 to 2016, more than 75% of all human vector-borne disease cases in the United States of America (USA) were caused by tick-borne pathogens [1]. Distribution and range changes of medically relevant tick species, such as *Ixodes scapularis* Say, *Haemaphysalis longicornis* Neumann, *Amblyomma americanum* Linnaeus, and *Amblyomma maculatum* Koch, raise concerns about potential undetected disease transmission and increased public health risk [1,2,3,4]. Despite rising incidence for tick-borne disease in the USA, surveillance remains fragmented and often siloed [4], with most vector control funding in the southeastern USA supporting mosquito surveillance and abatement [5].

There has been recognition of the presence of medically important tick vectors and their public health significance in South Carolina (SC), but no effort to formalize state-wide surveillance. Prior work in the state has primarily focused on narrow objectives, with fewer studies incorporating longitudinal data collection [6,7,8,9,10]. In 1978, the Bureau of Laboratories at the South Carolina Department of Health and Environmental Control conducted a study on spotted fever group *Rickettsia* (SFGR) tick species distribution and prevalence, and epidemiology in response to a Rocky Mountain Spotted Fever (RMSF) outbreak [9]. Public submissions for this effort yielded over 20,000 ticks collected during a 7-year period, with *Dermacentor variabilis* (Say)*, Am. americanum, Am. maculatum, Rhiphicephalus sanguineus* (Latreille)*,* and *Ix. scapularis* reported as the most common species submitted [11]. Another study from 1990–95 showed high *Am. americaum* abundance in the state [8].

Several multi-state cross-sectional studies were performed in the southern USA which included specimens from SC. One study evaluated several *Am. americanum* for *Ehrlichia* spp. and did not find any positives [12]. Another study tested *Am. americanum* ticks from multiple states and identified a single *Borrelia lonestari* positive tick and 45.6% *R. amblyommatis* prevalence in ticks collected from Hunting Island, SC [13], while sampling of ectoparasite presence and pathogen infection rates in free-roaming and captive animals in two SC zoological parks identified an *Anaplasma phagocytophilum* positive *Ix. scapularis* pool [14]. A multi-state study that included 11 specimens collected in SC found no evidence of *B. burgdorferi* in collected *Am. americanum* (a controversial debate at the time) [15]. A more recent study found no evidence of *B. miyamotoi* in *Ix. scapularis* ticks collected in SC [16].

Reported human tick-borne disease incidence in SC shows rising case burdens, similar to national reports but without a high Lyme disease burden. Between 2013 and 2017, 74% of human cases of vector-borne disease were caused by tick-borne pathogens. Approximately half (47%) of SC human cases were caused by spotted fever group rickettsioses (SFGR) [17,18]. According to publicly available data from the SC Department of Public Health, incidence of Lyme disease in the SC Lowcountry, the southernmost public health region of the state, has risen from 1.1 per 100,000 persons (2014–18) to 4.1 cases per 100,000 persons (2019–23) (SC DPH, 2025). Even with a rise in reported cases, these data categorize SC as a low-incidence Lyme disease state [19].

In addition to tick and pathogen surveillance gaps, clinicians and medical providers face challenges in tick-borne disease diagnosis—a global challenge not limited to our state. The non-specific clinical presentation, variable provider awareness of regional tick-borne disease risks, and poor species-specific laboratory testing can lead to delayed diagnosis and underestimation of disease burden [20,21,22,23]. Ultimately, this may reduce the uptake of public prevention measures, and yields little data to support expansion of surveillance, tick intervention work, or policy efforts to address tick-borne disease threats.

This paper describes the establishment of a tick surveillance program for the state of South Carolina spearheaded by the University of South Carolina (USC) and the South Carolina Department of Public Health (SC DPH, formerly SC DHEC). Joint efforts between academic institutions and local or state health departments have demonstrated regional successes in standardization of tick collection, real-time data sharing, and increased program capacity in the northeast and Pacific Coast states; however, tailoring these approaches to southeastern USA’s tick ecologies has yet to be performed [24,25]. South Carolina’s tick program initial goals were to: (1) establish a statewide collaborative network to conduct tick-borne disease research, (2) describe tick-borne pathogen distribution using active surveillance, (3) use these data to identify regions of increased tick-borne disease risk, and (4) improve tick-borne disease data quality at the agency, state, and national level.

## 2. Materials and Methods

### 2.1. Collaboration

Melissa Nolan (Associate Professor, USC) and Christopher Evans (State Entomologist, SC DPH) worked closely on project conceptualization, collection methods, scientific and logistical planning for pathogen testing, and data reporting for this effort. Former pre-doctoral student, Kyndall Dye-Braumuller, led field collections, morphological identification, tick processing, and provided data management and administrative support. Early in implementation, joint project meetings were held to discuss standardization of collection methods, data management, and logistics.

Both sites recruited volunteers for tick collecting through public advertisement (SC DPH) and student recruitment (USC). Undergraduate, graduate, and medical students were recruited through posted and circulated advertisements via the USC Office of Undergraduate Affairs. Student volunteers (n = 7) were offered course credit (e.g., independent study, honors thesis or masters practicum hours) and/or graduate school letters of recommendation to ensure benefit to the trainee. Interns, experiential learning opportunities or volunteer positions were offered throughout the spring, summer and/or fall semesters, with students asked to commit to one full academic semester. Students were trained on CDC collection methods by USC and SC DPH staff. A shared collection calendar was made with input from both teams. SC DPH-collected ticks were stored at the state medical entomology lab and transferred to USC for processing at year’s end.

Systematic collection of questing ticks was made possible through collaboration with the South Carolina State Park Service—a state-supported organization that manages and protects >90,000 acres of natural resources. Early phone calls with the state park service’s resource management office established the importance and need for routine tick sampling on public land. In December 2019, a state park research permit was secured for collecting ticks (permit #N-1-20). Due to the COVID-19 pandemic shutdown, state parks were closed for one month (April 2020); to continue tick collections, collaborations with the South Carolina Audubon Society provided alternative sampling locations for this period.

Convenience sampling of host-attached ticks from animal shelters was initiated by USC personnel who created a list of potential shelter collaborators located in key geographic regions. Shelters were ‘cold called’ by students and asked to participate in convenience sampling. Students traveled to interested shelters to provide collection supplies and answer questions. Monthly follow-up calls with each shelter were conducted to assess the need for additional collection supplies and/or coordinate sample transfer to USC.

### 2.2. Tick Collections

Ten sites were chosen based on documented tick–human contact and to make sure all four SC public health regions (Upstate, Midlands, Pee Dee, Lowcountry) were represented. At least two parks for each public health region were sampled approximately every other week to establish baseline tick species composition, distribution, and density: Paris Mountain and Caesars Head (Upstate); Kings Mountain, Sesquicentennial, and Dreher Island (Midlands); Woods Bay and Myrtle Beach (Pee Dee); and McAlhany Nature Preserve, Edisto Beach, Hunting Island, and Charleston County (Lowcountry).

**Figure 1:**
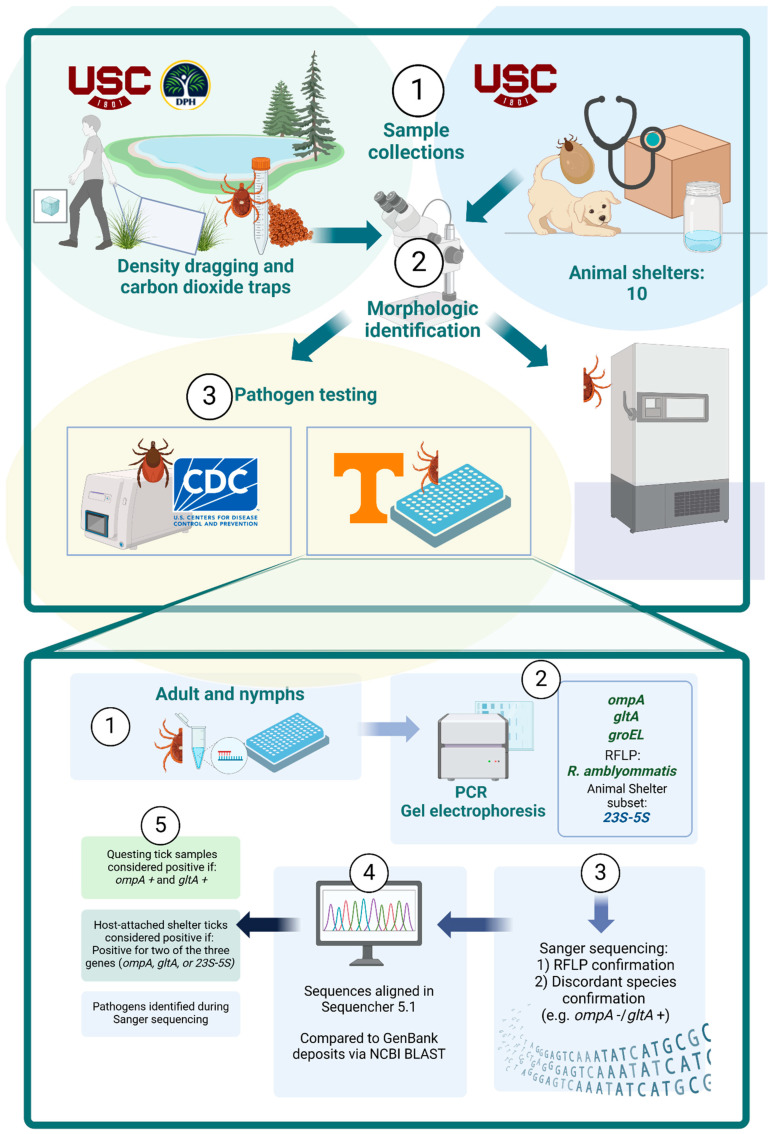
Schematic flow diagram of tick collection, processing, and testing.

Collections were conducted from March to October 2020 through active (tick dragging) and passive (CO_2_-baited traps and ticks found on the collectors) methods following CDC guidelines [26,27] (Figure 1). Drags were constructed from a 1.22 × 1.52 m white duck canvas attached to a 1.22 m wooden dowel, with 6 small zinc washers as weights on the bottom [26,27]. Dragging was conducted along nature trails in parks; each site was dragged for 30 min and drags were checked for ticks every 30 s; this ensured the recommended density-sampling surface area for host-seeking ticks was sampled at every collection site [26,27]. Tick collection teams of two students (graduate and undergraduates paired together) were deployed for each collection. Ticks were removed from the drag cloth and placed in a labeled vial filled with 75% ethanol. Traps baited with CO_2_ consisted of 0.61 m^2^ white muslin squares with 0.5–1 kg of dry ice placed directly in the center of the cloth square, and the cloth was set off nature trails in leaf litter or grass with 10 traps placed per collection. No adhesive was used to trap ticks; traps were left undisturbed for 1.5 to 2 h in the environment. Ticks were removed from the trap cloth and placed in 75% ethanol vials if there were 10 or fewer ticks left on the trap and folded into a plastic zip bag if there were over 10 ticks on the trap. Plastic bags with live ticks were placed in a −20 °C freezer overnight and dead ticks were then transferred to vials with 75% ethanol. Any ticks found on the collectors’ body were placed in vials and identified as “on body” or “biting”.

Ten animal humane shelters across the four public health regions agreed to collect ticks from stray dogs brought into their facilities from July to October 2020. Since tick removal was a part of routine animal veterinary health checks, this study was determined to be exempt of animal care research. Labeled 250 mL glass jars filled with 15 mL 75% ethanol were given to each animal shelter at the beginning of each month, and specimen-filled jars were returned for analysis. Collected glass jars contained a month’s worth of collected ticks from all animals brought into the shelter during that period. Contact with collaborating animal shelters was maintained monthly to answer questions and maintain active shelter participation.

### 2.3. Tick Processing

Within one week of collection, all ticks were identified to species, sex, life stage, and engorgement status. Morphological identifications were conducted with multiple dichotomous keys [28,29,30,31,32,33,34]. Suspected *Haemaphysalis longicornis* ticks were sent to the United States Department of Agriculture National Veterinary Services Laboratories (USDA NVSL) in Ames, Iowa for morphological confirmation, as these represented the first specimens of this invasive tick in South Carolina.

After taxonomic identification, flat/unfed adult *Ixodes* ticks were organized and shipped to the CDC Bacterial Diseases Branch, Division of Vector-Borne Diseases in Fort Collins, CO by USC as a single, statewide submission. Due to the COVID-19 pandemic, Dr. Nolan’s molecular laboratory was elicited for pandemic response testing, requiring an unanticipated need to collaborate with another medical entomology diagnostic laboratory for tick-testing support. Metastriate ticks were submitted to the Medical and Veterinary Entomology Laboratory at the University of Tennessee Knoxville (MVE UTK), led by Dr. Rebecca Trout-Fryxell.

### 2.4. Pathogen Testing and Sequencing

All adult host-seeking, unengorged *Ixodes* spp. were submitted to CDC for pathogen testing using real-time PCR to detect presence of *Borrelia* spp. and *Anaplasma* spp. Please note, ticks were not tested for *Babesia microti* due to a lack of prior molecular detection in southeastern USA *Ixodes* spp. ticks [35]. Methodologies used for CDC pathogen testing are described elsewhere [26,36]. *Ixodes* spp. collected from animal shelters (engorged) were tested at MVE UTK, using the methods described below. Specimens were bisected longitudinally with a sterilized scalpel blade; one half was stored in 80% ethanol as a voucher specimen, and the other half underwent nucleic acid extraction for pathogen testing. Ticks were individually homogenized in lysis buffer using a TissueLyser (Qiagen, Hilden, Germany). Total DNA was then extracted using the QIAamp 96 DNA Kit on a QIAcube HT (Qiagen, Hilden, Germany) according to the manufacturer’s protocol.

After extraction, specimens were pooled (up to 15 ticks per pool) by collection, species, sex, and life stage (adult and nymph). Standard PCR was used to screen each tick for up to three SFGR genes. All metastriate ticks were screened for the first SFGR gene, outer membrane protein gene *ompA*, using previously published methods [37]. All metastriate ticks were screened for a second SFGR gene, citrate synthase gene *gltA*, which was amplified through standard PCR. Reactions of 30 µL reaction volume were set up as follows: 15 µL DreamTaq^TM^ Hot Start Green PCR Master Mix (2X) (Thermo Fisher Scientific, Waltham, MA, USA), 11 µL nuclease-free water, 2 µL of sample DNA, and 1 µL of each 0.25 µM forward (Rr CS.372 TTT GTA GCT CTT CTC ATC CTA TGG C) and reverse (Rr CS.989 CCC AAG TTC CTT TAA TAC TTC TTT GC) primers [38]. Conditions were set as described by Kollars and Kengluecha (2001) [38] in a Veriti 96-Well Thermacycler (Thermo Fisher Scientific, Waltham, MA, USA). All PCR products were identified via gel electrophoresis (1.5% agarose gel: 1xTAE buffer stained with ethidium bromide for 1.5 h at 100 V).

A third SFGR gene, intergenic spacer gene *23S-5S*, was used for standard PCR in a subset of blood-fed and engorged ticks submitted from animal shelters. This was chosen to help clean up PCR results from the *ompA* and *gltA* screening as bands are difficult to read from engorged ticks. Reactions of 20 µL total volume were set up as follows: 10 µL DreamTaq^TM^ Hot Start Clear PCR Master Mix (2X), 6 µL nuclease-free water, 2 µL template DNA, and 1 µL of each 0.5 µM forward (RCK/23-5-F GAT AGG TCG GGT GTG GAA GCA C) and reverse (RCK/23-5-R GGG ATG GGA TCG TGT GTT TCA C) primers [39]. Conditions were set as described by Jado et al. (2006) [39]. For quality control, we used a *R. parkeri*-positive tick (positive control) and two negative controls: (1) no template control of nuclease-free water and (2) a confirmed *Rickettsia*-negative tick. Gel electrophoresis was conducted to identify products (1.5% agarose gel: 1xTAE buffer stained with ethidium bromide for 1.5 h at 100 V).

Due to the anticipated heavy burden of endosymbiotic *Rickettsia amblyommatis* in collected *Am. americanum* ticks, a restriction fragment length polymorphism assay (RFLP assay) was conducted to specifically screen for *R. amblyommatis* in this individual tick species [40]. Detailed screening methods for RFLP assay methods are also published [37]. A subset of 38 tick samples with positive *Rickettsia* amplicons were selected for further sequencing based on the following criteria: (1) when the RFLP suggested a different species than *R. amblyommatis* (n = 12), or (2) when a tick species other than *Am. americanum* screened positive for *gltA* and negative for *ompA* (n = 26).

Nested PCR reactions were conducted to detect *Ehrlichia* and *Anaplasma* spp. through screening for the conserved heat shock gene *groEL*. Detailed primers, reaction makeup, and conditions are described elsewhere [41,42]. Sequencing was attempted for all positive *groEL* amplicons (n = 37). Positive amplicons based on the defined criteria were sent to Eurofins Genomics (Louisville, KY, USA) for bi-directional Sanger sequencing. Resulting sequences of 376 to 378 bp lengths were aligned in Sequencher 5.1 (Gene Codes Corporation, Ann Arbor, MI, USA) and compared to GenBank deposits via NCBI Basic Local Alignment Search Tool (BLAST version 5.1) using default conditions [43,44].

To account for bacterial co-infection [45] and to improve testing sensitivity, individual ticks or pooled samples were considered positive if at least two genes were detected. For questing ticks, this meant detection of both *ompA* and *gltA,* and for animal shelter ticks, this meant detection of two of the three genes (*ompA*, *gltA*, or *23S-5S*).

### 2.5. Tick and Pathogen Distribution

For questing ticks, tick density and pathogen results were geocoded by collection site coordinates. A Level IV ecoregion (United States Environmental Protection Agency, US EPA) layer was added to these maps from the ArcGIS Living Atlas. Per shelter report, all animals originated from South Carolina, so ticks from shelters were geocoded to the originating county. The exact location of tick-infested stray animal collection was not available for analysis. Pathogen, tick species, and collection data were aggregated by location and displayed using choropleth imagery to represent density; spatial density was calculated using ArcGIS Pro’s density analysis tool. Maps were produced using ArcGIS Pro 3.1.3 (ESRI Corp, Redlands, CA, USA).

## 3. Results

Pathogen testing results were analyzed, cleaned, and reported to ArboNET, the national arbovirus surveillance system. Established in response to West Nile virus, ArboNET is managed by the CDC and state health departments; it was expanded in 2018 to include tick surveillance [24]. A total of 4520 ticks were collected from state parks (questing) and shelters (host-attached) (Table 1). Among questing ticks, five species were identified, comprising 3674 ticks: *Am. americanum* (98.1%), *Ix. scapularis* (1.4%), *Ix. keiransi* (<0.01%)*, De. variabilis* (<0.01%)*,* and *Am. maculatum* (<0.01%). All motile life stages were collected; the majority were adults (40.7%), followed by larvae (31.0%), and nymphs (28.3%). More female (56.6%) than male ticks (43.4%) were collected. Carbon dioxide traps yielded the greatest number of ticks (n = 1802), followed closely by tick drags (n = 1739), and crawling on or biting collectors’ bodies (n = 132). Ticks found attached to collector’s skin were identified within a few hours of a collection effort, and were not engorged. Tick dragging methods yielded slightly higher species diversity than carbon dioxide traps (5 species vs. 4 species).

For animal shelter ticks, eight species (n = 846) were identified, with 41% classified as unknown due to integument damage (could not make a morphological identification). Species distribution was: *Am. maculatum* (20.9%), *De. variabilis* (17.4%), *Am. americanum* (15.6%), *Ix. scapularis* (3.1%), *Ix. keiransi* Neumann (0.8%), *Rh. sanguineus* (0.6%), *Ixodes* spp. (0.5%), *Ha. longicornis* (0.2%), and *Ha. leporispalustris* (Packard) (0.2%). Host animal species and travel history from shelters were not documented. Anecdotally, veterinary staff reported most ticks were collected from stray dogs, with few from feral cats. Most ticks from animal shelters were adults, with females representing 51%. Ticks collected from animal shelters were both flat (44%) and fed (engorged or somewhat engorged, 56%).

### 3.1. Pathogen Testing: Metastriate Ticks

A total of 1303 ticks were at MVE UTK. Five hundred and thirty-nine (n = 539) *Am. americanum* were tested for both *ompA* and *gltA* genes (*Rickettsia* spp. genes—97.6% positive); ten of those ticks were screened for the *23S-5S* gene, and all ten were positive for both the *ompA/23S-5S* and *gltA/23S-5S* combinations (*Rickettsia* spp. genes). From the SFGR-positive ticks, 512 were confirmed *R. amblyommatis*-positive by RFLP. A total of 966 *Am. americanum* were tested for the *groEL* gene (1.9% positive), indicating presence of either *Ehrlichia* or *Anaplasma* spp. bacteria. *Amblyomma americanum* was the only species from parks that tested positive for both *Rickettsia* spp. and *Ehrlichia* or *Anaplasma* spp.

*Amblyomma americanum* ticks submitted from animal shelters were the only tick species with confirmed *R. amblyommatis* presence (30.8% were confirmed by RFLP), and *Ehrlichia* or *Anaplasma* spp. (2.6% were positive for the *groEL* gene). Among the 88 *Am. maculatum* ticks tested, only one was collected from a state park; this specimen was negative for *Rickettsia, Ehrlichia*, and *Anaplasma* spp. Of the remaining 87 ticks collected from animal shelters, 40 (87.0%) tested positive for the *ompA*/*23S–5S* gene combination, and 45 (97.8%) tested positive for *gltA/23S–5S*. Nearly half (47.1%) of ticks tested for both *ompA* and *gltA* were positive. None of the SFGR gene-positive ticks were *R. amblyommatis*-positive. Eighteen (20.7%) *Am. maculatum* ticks from animal shelters were positive for the *groEL* gene, indicating possible infection with *Ehrlichia* or *Anaplasma* spp.

All seven questing *De. variabilis* ticks collected from state parks were negative for *Rickettsia, Ehrlichia*, and *Anaplasma* spp. Among the 86 *De. variabilis* ticks from animal shelters, 12 (14.0%) tested positive for the *ompA/gltA* gene combination. Of 70 tested for *ompA/23S–5S* and *gltA/23S–5S*, 8 (11.4%) and 13 (18.6%) were positive, respectively. None of the SFGR-positive ticks were positive for *R. amblyommatis* or the *groEL* gene.

Of the five *Rh. sanguineus* ticks (shelter collected only), fewer than half were positive for SFGR genes, with no evidence of *R. amblyommatis*. One tick tested positive for the *groEL* gene, suggesting infection with *Ehrlichia* or *Anaplasma* spp. Neither of the two *Ha. leporispalustris* ticks tested from animal shelters were positive. The two *Ha. longicornis* collected ticks were not pathogen tested as they were preserved as voucher specimens by the USDA NVSL.

### 3.2. Pathogen Testing: Ixodes Ticks

Of the flat/unfed adult *Ixodes* spp. tested at the CDC (n = 45), two *Ix. keiransi* ticks were positive: one for an unknown *Borrelia* spp. and *B. burgdorferi* sensu stricto and one for *Anaplasma phagocytophilum* (Table 2). All *Ix. scapularis* ticks were negative for *Borrelia* spp. and *Anaplasma phagocytophilum*; however, *Ixodes scapularis* collected from animal shelters consistently tested positive for SFGR genes, with over 70% positive for all three gene combinations and none were positive for *R. amblyommatis*. Two (9.1%) *Ix. scapularis* tested positive for the *groEL* gene. All *Ix. keiransi* (100%) were positive for the three SFGR gene combinations and none were positive for *R. amblyommatis* or *groEL*.

### 3.3. Sanger Sequencing

Six *Am. americanum*, two *De. variabilis*, and one *Am. maculatum* tick aligned with *R. amblyommatis* (percent alignment ranging within 99.45–100% to KY273595 or KJ796417) (Table 3). Seven *De. variabilis*, five *Am. maculatum*, and three *Am. americanum* ticks showed 98.63–100% alignment with *R. parkeri* (KJ796435 or MG574939). Three *Am. maculatum* and two *De. variabilis* ticks had 100% alignment with *Candidatus* Rickettsia andeanae (KT153033); two *Am. maculatum* ticks showed 97.6–98.63% alignment with *Rickettsia montanensis* (KJ796427). Finally, one *Am. maculatum* and one *De. variabilis* tick showed 100% alignment with *Rickettsia asembonenesis* (OR523793).

One *Am. americanum* tick had 98–99% alignment with *Anaplasma odocoilei* (JX876642), and one *Ix. scapularis* had 98% alignment with *An. phagocytophilum* (MG570466) (Table 3). Five *Am. americanum* ticks had 99–100% alignment with *Ehrlichia ewingii* (KJ907744 or AF195273), and one *Ix. scapularis* had 99% alignment with *E. ewingii* (KJ907744). Three *Am. americanum* ticks had 98–99% alignment with *E. chaffeensis* (KJ907753), and one *Am. maculatum* had 91% alignment with *E. chaffeensis* (KJ907753). Three *Am. americanum* ticks had 91–100% alignment with Panola Mountain *Ehrlichia* (HQ658904). Lastly, one *Am. maculatum* tick had 96% alignment with *Ehrlichia* sp. strain Córdoba (KY425416).

### 3.4. Seasonality

**Figure 2:**
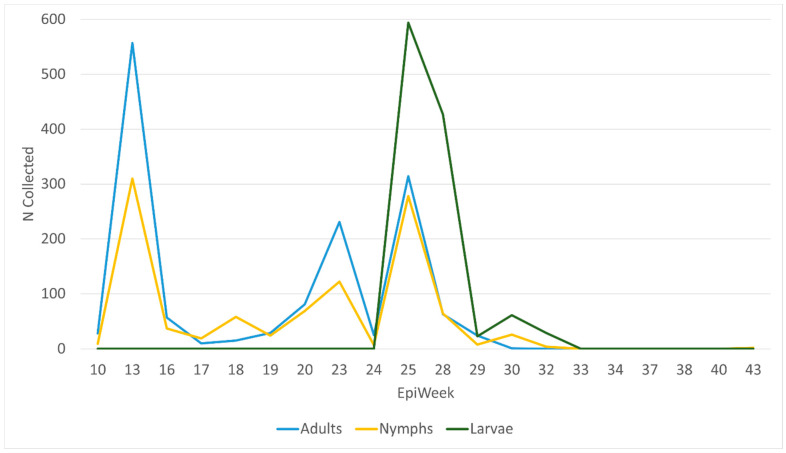
Frequency of *Amblyomma americanum* collected by EpiWeek in SC State Parks, by life stage.

Three collection peaks were observed: EpiWeek 13 (22–28 March 2020), EpiWeek 23 (31 May–6 June 2020), and EpiWeek 25 (14–20 June 2020) (Figure 2). The EpiWeek 25 peak yielded the highest volume of *Am. americanum* ticks, with approximately 1200 ticks collected. Adult *Am. americanum* comprised most ticks collected until EpiWeek 25, when we observed a shift to large numbers of larvae collected. Throughout the collection period, nymphal activity peaks typically followed adult patterns. *Ixodes scapularis* peaked at EpiWeek 13 (22–28 March 2020) (Figure S1). All other species’ seasonality cannot be accurately interpreted due to low collection counts.

### 3.5. Geographic Distribution

**Figure 3:**
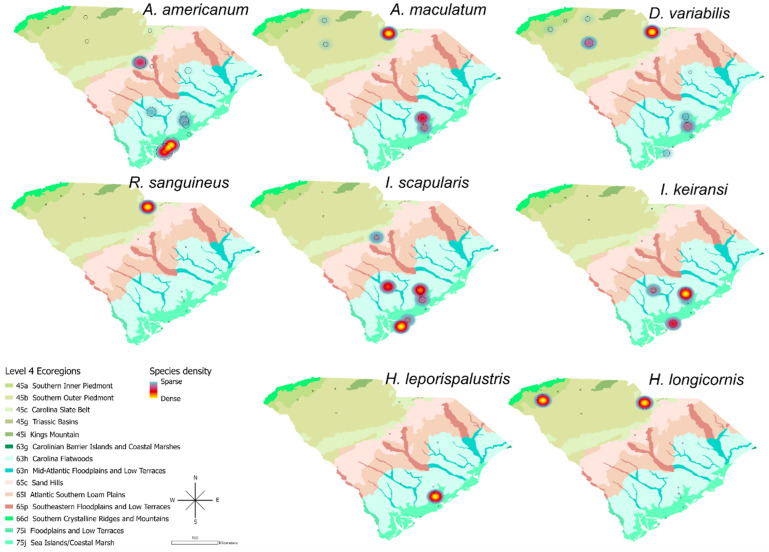
Map of species distribution spatial density by ecoregions.

The highest volume of collected ticks was in the southern coastal area, where a large proportion of *Am. americanum*, *Ix. scapularis*, and *Ix. keiransi* were collected (Figure 3). Most of the ticks from the Upstate or Pee Dee regions were submitted from animal shelters (Figure S2). In contrast, the northern part of the state had a greater volume of *Am. maculatum, De. variabilis*, *Rh. sanguineus*, and *Ha. longicornis.*

**Figure 4:**
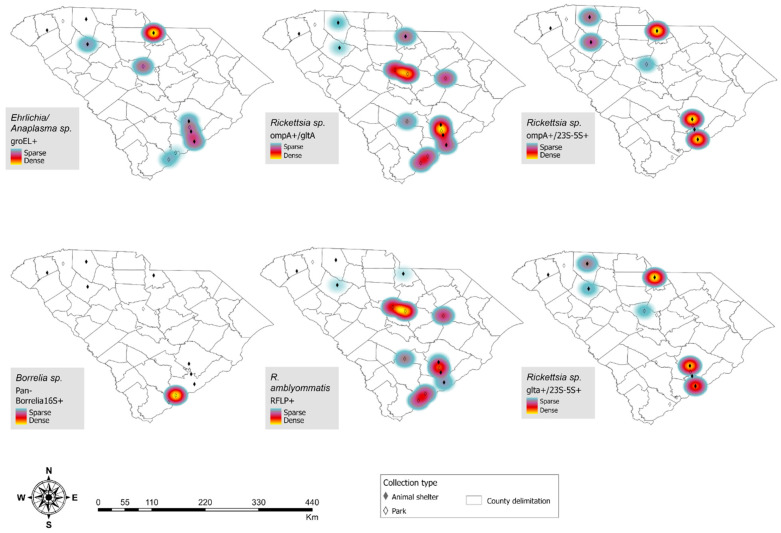
Geographic distribution of pathogen test results, including density of positives for each collection site (animal shelters and parks).

SFGR *ompA*+*/gltA*+ distribution and *R. amblyommatis*-positive tick geographies overlapped, with diffuse presence across the state. *Ehrlichia* and *Anaplasma* spp.-positive ticks were distributed along the northern and coastal regions, and one *B. burgdorferi* sensu stricto-positive tick was from a state park in the southern coastal region (Figure 4).

## 4. Discussion

This surveillance approach established a scalable, statewide, interdisciplinary network for tick research, integrating trainees into field and laboratory workflows to expand capacity and support workforce development. Sequencing confirmed the presence of multiple Rickettsia, Ehrlichia, and *Anaplasma* spp. and only one tick infected with *B. burgdorferi s.s*. Pathogen results from Am. americanum ticks revealed a high R. amblyommatis infection rate (53.2%), in line with a previous report from the SC coast [13], and a trend we also observed in ticks collected between 2021 and 2022 [46]. *R. amblyommatis* is widely distributed across the southeastern USA [47]. Its role in human disease remains debated, with no direct evidence supporting clinical pathogenicity beyond laboratory models [23,47,48,49]. *Candidatus R. andeanae*, also detected in the region, has no known human pathogenicity though may play an exclusionary role for other SFGR species in ticks [50,51,52]. Infection was not detected in any of the 2020 questing *De. variabilis*, *Am. maculatum*, or *Ix. scapularis* ticks and only two *Ix. keiransi* ticks were positive (one for *B. burgdorferi s.s.* and one for *A. phagocytophilum*).

*R. parkeri*, considered the most pathogenic species identified, is thought to be the second most prevalent tick-borne SFG rickettsiae in the Americas and is widely distributed across the southeastern USA [50,51,53,54]. Two *Am. maculatum* were positive for *R. montanensis*, a species historically considered non-pathogenic but increasingly implicated in misdiagnosed cases in the mid-Atlantic and southeastern USA [49,55,56], and previously reported in *Am. maculatum* in this region [54,55,57,58].

In animal shelter-submitted ticks, every tested species was positive for a rickettsial agent except Ha. leporispalustris. Ix. scapularis was positive for all three rickettsial genes tested, and approximately 9% were positive for either *Ehrlichia* or *Anaplasma* spp. *Ix. keiransi* was positive for all three SFGR genes tested, whereas De. variabilis had a lower SFGR-positive percentage than anticipated. Am. maculatum ticks had 20.7% positivity for *Ehrlichia* or *Anaplasma* spp. Notably, one *De. variabilis* and one *Am. maculatum* amplified sequences with 100% alignment to *R. asembonensis*, a flea-associated species rarely reported in ticks [59,60,61]. Although previously considered non-pathogenic, emerging evidence suggests potential involvement in human and animal disease [62,63,64,65,66]. Detection in these ticks may reflect co-feeding of infected fleas and naïve ticks on shared hosts, though evidence for this mechanism remains limited [61,67,68].

Most human ehrlichiosis cases in the USA are caused by *E. chaffeensis*, *E. ewingii*, *E. muris eauclairensis*, and, rarely, Panola Mountain *Ehrlichia* [69,70,71], primarily transmitted by *Am. americanum* [23,43,54,72]. The increasing incidence of ehrlichiosis nationwide underscores the clinical relevance of detecting multiple *Ehrlichia* spp. in ticks collected in SC [69,73]. Human anaplasmosis incidence is also increasing annually [74,75]. Although *Ix. scapularis* is the primary vector, *A. phagocytophilum* has also been reported in *Am. americanum* [54], suggesting that inclusion of multiple tick species in surveillance may help identify additional transmission pathways. South Carolina has reported 26 cases of undetermined ehrlichiosis/anaplasmosis, with the highest number occurring in southern coastal counties, though the causative agents and tick species involved remain unknown [76].

Historically, SC has reported RMSF outbreaks associated with SFGR-infected ticks [9]; the majority of SFGR-positive ticks in this study were positive for *R. amblyommatis*, and none were infected with *R. rickettsii*. Previous outbreak investigations collected higher numbers of *De. variabilis*, the primary vector of *R. rickettsii*, whereas this study predominantly collected *Am. americanum* [6], which may reflect differences in sampling methods or shifts in tick distribution.

This program aimed to establish a sustainable surveillance network and generate pilot data to support expansion of tick-borne disease research, outreach, and education [77]. The South Carolina tick surveillance program is now recognized among state veterinarians, local health systems, the public, and academic partners as a resource for tick-borne disease expertise. Our sustained collaborations have positioned us to address emerging tick-related public health threats. Programmatically, we met key milestones by building capacity for tick-borne disease research, generating baseline data, and supporting training of a future medical entomology workforce.

The program has expanded from bimonthly collections at 10 sites to year-round surveillance at more than 45 sites, each sampled every six weeks. Resulting data have contributed to peer-reviewed publications, successful grant funding, methodological innovation, and increased policy engagement at state and national levels [46,78,79]. These data have also informed intervention studies using innovative approaches to detect, control, and mitigate the impact of tick-borne disease (studies are ongoing).

There were several important lessons learned throughout this process. Despite efforts to harmonize data collection, differences across sites led to time-intensive cleaning and reconciliation prior to analysis. Establishing a standardized set of shared data points a priori supports the generation of reliable, high-quality datasets. The volume of data generated is substantial, and participating sites should determine early how data will be stored, managed, and shared. For our work, Microsoft Excel served as the primary platform for data collection, allowing alignment with CDC data points for *Ixodes* submissions. As data volume increased, organization into three categories—(1) collection data, (2) pathogen testing results, and (3) aggregated summary data—by year of surveillance proved critical and required coordinated support from multiple team members.

Given that these data represent collections from a single year, findings should be interpreted cautiously. Shelters did not record the date of tick collection, host animal species or travel history, and samples were stored in a single container. Our primary goal was to engage and establish consistent collection with shelters, which we accomplished. Methods of collection since 2020 have been refined to include more detailed data collection and follow-up with shelters. Animal shelter sites are also prone to selection bias, since the number of ticks submitted may not correspond to true regional tick abundance or distribution. Variability in staff engagement and interest likely influenced participation levels, with some shelters submitting substantially more specimens than others.

## 5. Conclusions

As Burgdorfer et al. emphasized in their study of RMSF in South Carolina, “…only through educational programs and availability of tick examination services, vigorously pursued, can a significant decrease be brought about in the incidence and mortality from this disease” [6]. Data-driven public health preparedness is a fulcrum of adequately addressing emerging and re-emerging infectious diseases. Together, these findings demonstrate that coordinated surveillance can generate region-specific risk profiles and broader insights into evolving tick-borne disease ecology using a cooperative framework and support national goals in the fight against vector-borne disease.

## Figures and Tables

**Table 1:** Tick species collected by sex, collection method, and life stage.

Ticks Collected from South Carolina Parks (n = 3674)										
	Life Stage			Sex (Adults Only)		Collection Method				Total (%)
	Adult	Nymph	Larva	Male	Female	Drag	Trap	Body *	Bite **	
*Amblyomma americanum*	1435	1037	1134	618	817	1690	1784	109	23	3606 (98.1)
*Amblyomma maculatum*	1	0	0	1	0	1	0	0	0	1 (0)
*Ixodes scapularis*	48	0	5	22	26	40	12	1	0	53 (1.4)
*Ixodes keiransi*	7	0	0	4	3	2	5	0	0	7 (0.2)
*Dermacentor variabilis*	7	0	0	5	2	6	1	0	0	7 (0.2)
Ticks submitted from South Carolina animal shelters (n = 846)										
	Life stage			Sex (adults only)		Fed status				Total (%)
	Adult	Nymph	Larva	Male	Female	Fed		Flat		
*Amblyomma americanum*	80	52	0	24	56	83		49		132 (15.6)
*Amblyomma maculatum*	162	15	0	104	58	33		144		177 (20.9)
*Ixodes scapularis*	26	0	0	6	20	8		18		26 (3.1)
*Ixodes keiransi*	7	0	0	0	7	7		0		7 (0.8)
*Ixodes* unknown spp.	4	0	0	0	4	4		0		4 (0.5)
*Dermacentor variabilis*	147	0	0	76	71	43		104		147 (17.4)
*Haemaphysalis longicornis*	2	0	0	0	2	2		0		2 (0.2)
*Haemaphysalis leporispalustris*	2	0	0	0	2	0		2		2 (0.2)
*Rhipicephalus sanguineus*	2	3	0	0	2	0		5		5 (0.6)
Unknown spp. ^†^	2	86	256	0	0	296		48		344 (40.7)

^†^ Adult unknown species were too crushed or missing too many body parts to be confidently morphologically identified to species or sex. * Ticks found on the collectors’ bodies, but not biting, were counted as ‘body’. ** Ticks found on the collectors’ bodies actively biting were counted as ‘bite’. These were not included in the ‘body’ tick count.

**Table 2:** Pathogens detected in tested ticks, by species.

Ticks Collected from South Carolina State Parks N+/N Tested (%)								
	Tested at MVE UTK					Tested at CDC Bacterial Diseases Branch		
	*Rickettsia* spp.^ ◊^				*Ehrlichia/Anaplasma* spp.	*Borrelia* spp.	*Borrelia burgdorferi* ss (Bbss)	*Anaplasma phagocytophilum* (Ap)
	*ompA+* and *gltA+*	*ompA+* and *23S-5S+*	*gltA+* and *23S-5S+*	RFLP confirmed (*R. amblyommatis*)	*groEL+*	Pan-*Borrelia 16S+*	Pan-*Borrelia 16S+,* Bbss *fliD+,* and Bbss *oppA2+*	Ap *p44+* and Ap *msp4+*
*Amblyomma americanum*	526/539 (97.6)	10/10 (100)	10/10 (100)	512/962 (53.2)	18/966 (1.9)	-	-	-
*Amblyomma maculatum* ^†^	0/1 (0)	-	-	0/1 (0)	0/1 (0)	-	-	-
*Ixodes scapularis*	-	-	-	-	-	0/38 (0)	0/38 (0)	0/38 (0)
*Ixodes keiransi*	-	-	-	-	-	1/7 (14.3)	1/7 (14.3)	1/7 (14.3)
*Dermacentor variabilis* ^†^	0/7 (0)	-	-	0/7 (0)	0/7 (0)	-	-	-
Ticks submitted from South Carolina animal shelters * N+/N tested (%)								
	Tested at MVE UTK							
	*Rickettsia* spp.^ ◊^				*Ehrlichia/Anaplasma* spp.			
	*ompA+* and *gltA+*	*ompA+* and *23S-5S+*	*gltA+* and *23S-5S+*	RFLP confirmed (*R. amblyommatis*)	*groEL+*			
*Amblyomma americanum*	46/117 (39.3)	26/72 (36.1)	30/72 (41.7)	36/117 (30.8)	3/117 (2.6)			
*Amblyomma maculatum*	41/87 (47.1)	40/46 (87.0)	45/46 (97.8)	0/87 (0)	18/87 (20.7)			
*Ixodes scapularis*	18/22 (81.8)	7/10 (70.0)	9/10 (90.0)	0/22 (0)	2/22 (9.1)			
*Ixodes keiransi*	6/6 (100)	6/6 (100)	6/6 (100)	0/6 (0)	0/6 (0)			
*Ixodes* spp. unknown	4/4 (100)	4/4 (100)	4/4 (100)	0/4 (0)	0/4 (0)			
*Dermacentor variabilis*	12/86 (14.0)	8/70 (11.4)	13/70 (18.6)	0/86 (0)	0/86 (0)			
*Haemaphysalis leporispalustris* ^‡^	0/2 (0)	-	-	0/2 (0)	0/2 (0)			
*Rhipicephalus sanguineus*	2/5 (40.0)	2/5 (40.0)	1/5 (20.0)	0/5 (0)	1/5 (20.0)			

* Both *Haemaphysalis longicornis* ticks were sent to the National Veterinary Diagnostic Laboratory and retained as voucher specimens; these and all unknown species were not tested for pathogens. ^†^ *Amblyomma maculatum* and *Dermacentor variabilis* ticks collected in state parks were not tested for the *23S-5S* gene. ^‡^ *Haemaphysalis leporispalustris* ticks collected from animal shelters were not tested for the *23S-5S* gene. ^◊^ Testing of all three *Rickettsia* spp. genes was not available for all ticks. The algorithm for determining true positive ticks was if a tick tested positive for at least two of the three genes tested. This was to increase the sensitivity of the testing.

**Table 3:** Sequencing results from *Rickettsia gltA* or *23S-5S* positive ticks or *Ehrlichia/Anaplasma groEL*-positive ticks.

Pathogen Sequencing Archetype	Species	Sample ID	Collection Date	Collection Type	Engorgement Status	Life Stage	Sex	Species Identity	% Alignment and GenBank Accession Number	Positive on Other *Rickettsia* Gene Target Test
Sequencing results from *gltA* products	*Amblyomma americanum*	AAF0775	6/19/2020	park	flat	adult	F	*R. amblyommatis*	100%	N
									KY273595	
	*Amblyomma americanum*	AAF0886	7/10/2020	park	flat	adult	F	*R. amblyommatis*	100%	N
									KY273595	
	*Amblyomma americanum*	AAF0981 *	8/21/2020	animal	engorged	adult	F	*R. parkeri*	100%	N
									MG574939	
	*Amblyomma americanum*	AAM1033 ^†^	8/21/2020	animal	flat	adult	M	*R. amblyommatis*	100%	N
									KY273595	
	*Amblyomma americanum*	AAN1066 ^†^	8/21/2020	animal	engorged	nymph	I	*R. amblyommatis*	100%	Y
									KY273595	
	*Dermacentor variabilis*	DVF1010 *	8/21/2020	animal	engorged	adult	F	*R. parkeri*	100%	N
									MG574939	
	*Dermacentor variabilis*	DVF1012 *	8/21/2020	animal	engorged	adult	F	*R. parkeri*	100%	N
									MG574939	
	*Dermacentor variabilis*	DVF1013 *	8/21/2020	animal	engorged	adult	F	*R. amblyommatis*	99.62%	N
									KY273595	
	*Dermacentor variabilis*	DVF1054 ^†^	8/21/2020	animal	flat	adult	F	*R. parkeri*	100%	N
									MG574939	
	*Amblyomma americanum*	AAM1129 ^‡^	8/24/2020	animal	flat	adult	M	*R. parkeri*	100%	N
									MG574939	
	*Amblyomma americanum*	AAF1154 ^‡^	8/24/2020	animal	engorged	adult	F	*R. amblyommatis*	100%	Y
									KY273595	
	*Amblyomma maculatum*	AMF1160 ^‡^	8/24/2020	animal	flat	adult	F	*R. parkeri*	100%	Y
									MG574939	
	*Amblyomma maculatum*	AMF1295 ^‡^	8/24/2020	animal	engorged	adult	F	*R. parkeri*	100%	Y
									MG574939	
	*Amblyomma maculatum*	AMF1298 ^‡^	8/24/2020	animal	engorged	adult	F	*Ca.* R. andeanae	100%	Y
									KT153033	
	*Amblyomma maculatum*	AMM1173 ^‡^	8/24/2020	animal	flat	adult	M	*Ca.* R. andeanae	100%	Y
									KT153033	
	*Dermacentor variabilis*	DMF1214 ^‡^	8/24/2020	animal	engorged	adult	F	*R. parkeri*	100%	Y
									MG574939	
	*Dermacentor variabilis*	DVF1215 ^‡^	8/24/2020	animal	engorged	adult	F	*Ca.* R. andeanae	100%	Y
									KT153033	
	*Dermacentor variabilis*	DVF1223 ^‡^	8/24/2020	animal	engorged	adult	F	*R. amblyommatis*	99.79%	N
									KY273595	
	*Dermacentor variabilis*	DVM1227 ^‡^	8/24/2020	animal	flat	adult	M	*R. parkeri*	100%	Y
									MG574939	
	*Dermacentor variabilis*	DVM1232 ^‡^	8/24/2020	animal	flat	adult	M	*R. parkeri*	99.62%	N
									MG57439	
	*Dermacentor variabilis*	DVM1233 ^‡^	8/24/2020	animal	flat	adult	M	*R. parkeri*	100%	Y
									MG574939	
	*Dermacentor variabilis*	DVM1236 ^‡^	8/24/2020	animal	flat	adult	M	*Ca.* R. andeanae	100%	Y
									KT153033	
	*Amblyomma maculatum*	AMF1303 ^◊^	9/18/2020	animal	engorged	adult	F	*R. parkeri*	100%	Y
									MG574939	
	*Amblyomma maculatum*	AMM1082 ^◊^	9/18/2020	animal	flat	adult	M	*R. parkeri*	100%	Y
									MG574939	
	*Amblyomma maculatum*	AMM1090 ^◊^	9/18/2020	animal	flat	adult	M	*Ca.* R. andeanae	100%	Y
									KT153033	
Sequencing results from *23S-5S* products	*Amblyomma americanum*	AAF1300	8/11/2020	animal	engorged	adult	F	*R. amblyommatis*	100%	Y
									KJ796417	
	*Amblyomma americanum*	AAN1189 ^‡^	8/24/2020	animal	engorged	nymph	I	*R. parkeri*	98.63%	N
									KJ796435	
	*Amblyomma maculatum*	AMM1183 ^‡^	8/24/2020	animal	flat	adult	M	*R. montanensis*	98.63%	Y
									KJ796427	
	*Amblyomma maculatum*	AMF1299 ^‡^	8/24/2020	animal	engorged	adult	F	*R. amblyommatis*	99.45%	N
									KJ796417	
	*Amblyomma maculatum*	AMN1191 ^‡^	8/24/2020	animal	engorged	nymph	I	*R. asembonensis*	100%	N
									OR523793	
	*Dermacentor variabilis*	DVF1206 ^‡^	8/24/2020	animal	engorged	adult	F	*R. asembonensis*	100%	Y
									OR523793	
	*Amblyomma maculatum*	AMF1303	9/18/2020	animal	engorged	adult	F	*R. parkeri*	99.72%	Y
									KJ796435	
	*Amblyomma maculatum*	AMM1208	11/3/2020	animal	flat	adult	M	*R. montanensis*	97.60%	Y
									KJ796427	
Sequencing results from *groEL* products	*Amblyomma americanum*	AAN0012	3/6/2020	park	flat	nymph	I	*A. odocoilei*	98%	N/A
									JX876642	
	*Amblyomma americanum*	AAF0083	3/27/2020	park	flat	adult	F	*A. odocoilei*	99%	
									JX876642	
	*Amblyomma americanum*	AAF0105	3/27/2020	park	flat	adult	F	*E. ewingii*	99%	
									KJ907744	
	*Amblyomma americanum*	AAM0427	5/15/2020	park	flat	adult	M	*E. ewingii*	100%	
									KJ907744	
	*Amblyomma americanum*	AAM0485	6/1/2020	park	flat	adult	M	*E. chaffeensis*	99%	
									KJ907753	
	*Amblyomma americanum*	AAM0481	6/1/2020	park	flat	adult	M	*E. chaffeensis*	99%	
									KJ907753	
	*Amblyomma americanum*	AAN0543	6/1/2020	park	flat	nymph	I	Panola Mtn *Ehrlichia*	100%	
									HQ658904	
	*Amblyomma americanum*	AAM0553	6/1/2020	park	flat	adult	M	Panola Mtn *Ehrlichia*	91%	
									HQ658904	
	*Amblyomma americanum*	AAN0643	6/17/2020	park	flat	nymph	I	*A. odocoilei*	99%	
									JX876642	
	*Amblyomma americanum*	AAM0703	6/17/2020	park	flat	adult	M	*E. chaffeensis*	99.40%	
									KJ907753	
	*Amblyomma americanum*	AAF0818	7/10/2020	park	flat	adult	F	*E. chaffeensis*	98%	
									KJ907753	
	*Amblyomma americanum*	AAM0857	7/10/2020	park	flat	adult	M	Panola Mtn *Ehrlichia*	100%	
									HQ658904	
	*Amblyomma americanum*	AAF0976	8/21/2020	animal	engorged	adult	F	*E. ewingii*	99%	
									KJ907744	
	*Amblyomma americanum*	AAF1133	8/21/2020	animal	flat	adult	F	*E. ewingii*	99%	
									KJ907744	
	*Ixodes scapularis*	ISF1052 ^†^	8/21/2020	animal	engorged	adult	F	*E. ewingii*	99%	
									KJ907744	
	*Ixodes scapularis*	ISM1053 ^†^	8/21/2020	animal	flat	adult	M	*A. phagocytophilum*	98%	
									MG570466	
	*Amblyomma maculatum*	AMM1197	8/24/2020	animal	engorged	adult	M	*E. chaffeensis*	91%	
									KJ907753	
	*Amblyomma maculatum*	AMM1089	9/18/2020	animal	flat	adult	M	*Ehrlichia* Cordoba	96%	
									KY425416	

Matching symbols indicate which ticks were collected from the same animal shelter from the same date: * All collected from the same animal shelter during one month. ^†^ All collected from the same animal shelter during one month. ^‡^ All collected from the same animal shelter during one month. ^◊^ All collected from the same animal shelter during one month.

## Data Availability

The raw data supporting the conclusions of this article will be made available by the authors upon request.

## Supplementary Materials

The following supporting information can be downloaded at: https://www.mdpi.com/article/10.3390/insects17040414/s1, Figure S1: All tick species (except *A. americanum*) collected by EpiWeek in SC State Parks; Figure S2: Total tick collections distribution across animal shelter and state park collection sites.
