# Supplementary material for: Filling the Gap: Establishing a Statewide Tick and Tick-Borne Pathogen Surveillance Program
Source: Insects. 2026 Apr 12;17(4):414. doi: 10.3390/insects17040414 (PMC13116939; doi:10.3390/insects17040414)

Figure S1. All tick species (except *A. americanum*) collected by EpiWeek in SC State Parks

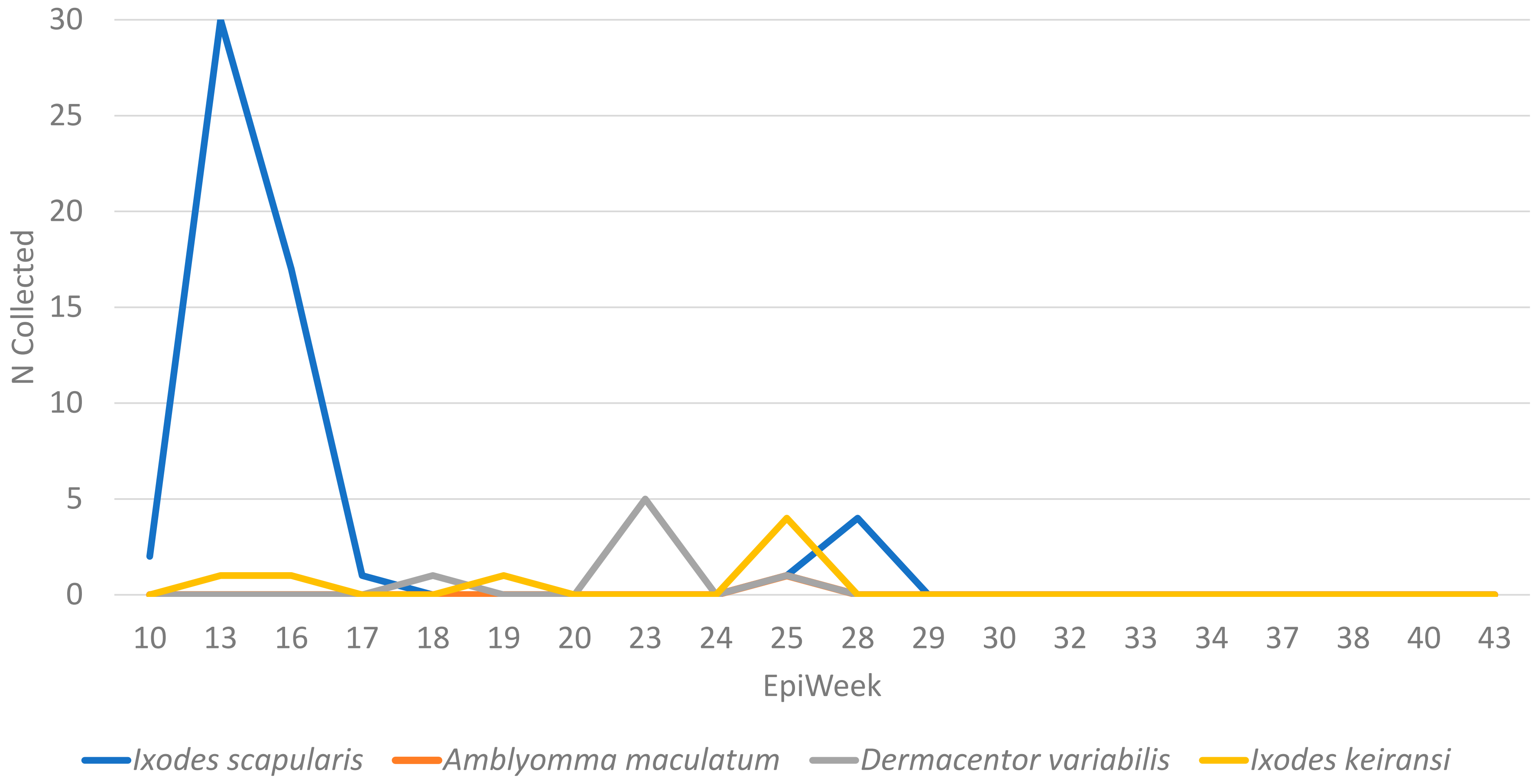

Figure S2. Total tick collections distribution across animal shelter and state park collection sites.

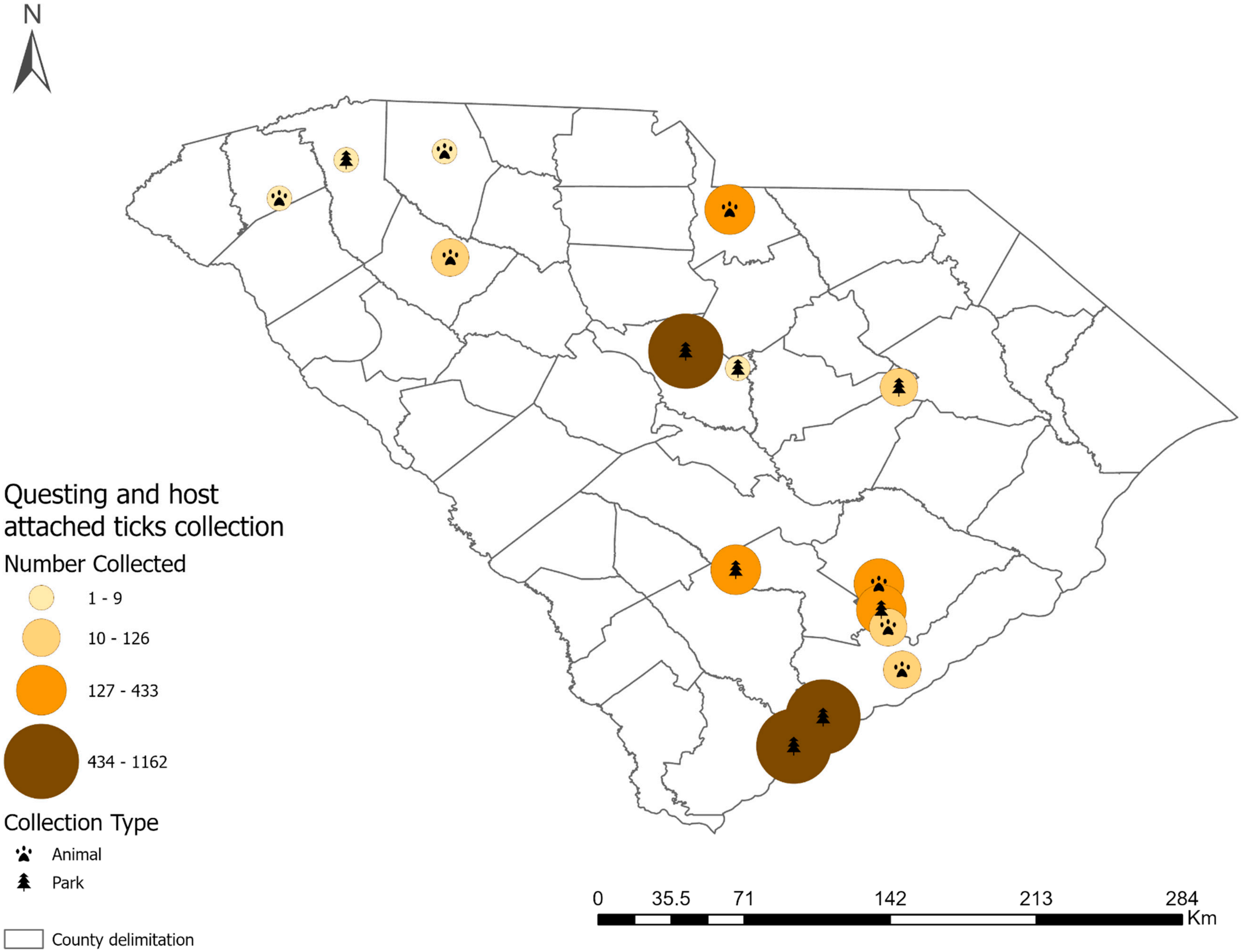

Supplement: Supplementary file 1 [file insects-17-00414-s001.zip › insects-4202503-supplementary.pdf]
